# Reviews and Book Notices

**Published:** 1878-01

**Authors:** 


### Reviews and Book Notices.

THE EAR: ITS ANATOMY, PHYSIOLOGY, AND DISEASES. By Charles H. Burnett, A.M., M.D.; Henry C. Lea, Philadelphia, publisher.

The author of this work may be congratulated on having accomplished the task of presenting to the profession, clearly and concisely, the present aspect of otology.

It is a more than ordinary pleasure to review a book in which there is so much to be commended and so little to be criticised, as in this.

Especially is this so in this case, since, until so recent a date, the field here covered was characterized by noticeable absence of any reliable or satisfactory information.

The anatomy and physiology, as here presented, give the reader the benefit of the most recent investigations in both, and prepare the way satisfactorily for the pathology, therapeutics, and surgery which follow.

In addition to imparting the latest reliable information, he has sought to utilize well-known facts, by showing their practical application—rather than to group together vague uncertainties, for the sake of supposed novelty. Where he is not convinced of the practical value of proposed methods of treating disease, it is frankly stated, and the reader is left to draw his own conclusions.

Regarding the non-suppurative inflammations, especially the chronic form, involving the middle ear, one could have wished that the author might have felt himself justified in speaking more confidently as to treatment and prognosis. Since these affections have been distinguished from so-called nervous deafness, and been assigned their proper place among diseases of the conducting apparatus, the results of treatment have been more satisfactory, but our prognosis still savors of the doubt that we have been so long accustomed to feel regarding them, and this has a tendency to restrain our efforts in their behalf.

Whilst, in some respects, the author's experience has seemed to differ from that of other aural surgeons, his position is so presented as to challenge respect for it from those who may feel that they must, though reluctantly, disagree with him as to some of the views held.

The article on suppurative affections gives additional urgency to the reasons so often adduced why they should not be neglected in the future, as they have been in the past, notwithstanding the disastrous, and sometimes fatal results, which have followed neglect or improper treatment of them.

The course of treatment advised is, in the main, wise and judicious, and is the result of intelligent observation of the effect of such treatment.

The section on deaf-mutes and partially deaf persons shows a step forward and in the right direction. It may safely be assumed, as stated, that the statistics regarding the deaf and dumb are greatly wanting in accuracy, and our conclusions having so long been drawn from fallacious premises, have been and must be erroneous.

The defects which prevent hearing, and thus produce muteness, are so much more frequently acquired than congenital, and are so often remediable, that it is to be hoped that greater efforts may, in future, be made to remove the cause of this affliction, instead of limiting our work to trials to reduce the resulting disadvantage, and to treat more frequently and more successfully the conditions on which deafness and consequent muteness depend.

The publisher of the work has issued it in a form worthy of a book that is destined to do so much good and so little harm.

S. J. J.

Public Health Reports and Papers, Vol. III. Presented at the meetings of the American Public Health Association in the years 1875–1876.

This is a collection of essays read before the American Public Health Association, at its annual meeting in Boston, September, 1876. They treat of various topics in which sanitarians are supposed to feel an interest. As might be expected, the comparative value of the different papers is very unequal—some of them being quite interesting, while others form the most commonplace kind of "padding." In this connection it may be observed that the bulk of the present volume is far less than that of its predecessors; and it is to be hoped that the future volumes may experience a still greater condensation. So far as the writers deal in descriptive effort, such as may be found in the essays which treat of the slaughter-house system of New York and Boston, their work is fairly well done. The statistical papers which, like Dr. Woodworth's, bring together a number of figures and estimates which are not easily accessible, are of value to those who desire such information. It is in the scientific and theoretical departments that the poverty of the volume is most conspicuous. It really seems hardly worth while to be at the expense of thus publishing scraps of science, some of it of doubtful quality, which can be found more recently and freshly set forth upon the pages of the numerous scientific periodicals which are found on the shelves of every student. It is really no compliment to the intellectual equipment of the average health official to suppose that he is in need of the tardy and intermittent supply of knowledge which filters through such a series of volumes. In fact, it is one of the misfortunes of such a publication that it addresses itself to no large constituency of readers. For the leaders of science it possesses very little worth; and for the mass of uninstructed readers its contents are not presented in a sufficiently available form. For example, the admirable discourse on Food, by Dr. Flint, contains nothing which is not well known to the majority of educated people. It is, therefore, hardly worth while to fill so much space with utterances which in this volume are practically withdrawn from the classes to which they might profitably be addressed. A public health association which would expend its energy in the emission and wide circulation of tracts of a popular character, like Dr. Flint's address, would accomplish far more good than can ever be effected by the annual publication of a single heavy volume which nobody will read.

It is not pleasant to be obliged to speak thus of work which is the offspring of the purest philanthropy the world has ever seen. To lie in idleness, and to hear only the salt sea-waves rippling over the pages of Dr. Gibson's breezy essay, would be far more agreeable; but if the feelings are allowed to run away with one's reason, there is an end of scientific progress. A book for which so much is claimed demands more than ordinary scrutiny.

As already stated, the best features of the volume are its descriptive portions. Its contributors have a keen eye for filth—a sharp nose for sewer-gas. But as for any attempt to penetrate to the deep underlying causes which control the origin and the prevalence of disease, it scarcely appears. Knowing little, apparently, of these great world-causes, and of the wonderful system of checks and counter-checks in which such causes play so conspicuous a part, these gentlemen very naturally turn for relief to artificial, human institutions. Ignorant of natural law, they would have us seek refuge under the wing of the immaculate police-justice, and they call loudly for fines and for legal processes, with which to "stamp out" disease. Some of our sanitarians, indeed, seem never to be thoroughly happy unless they can be hauling some one before the courts, and can parade a list of punishments for constructive offenses longer than the moral law. In pleasing contrast with this style is the excellent paper on Vaccination, by the distinguished President of the Association, Dr. Elisha Harris. The statesman-like way in which he treats this difficult subject is above praise, and stands in delightful opposition to the methods of the ordinary official. And yet Dr. Harris does not penetrate to the bottom of his subject, and fails to make clear the reasons which have hitherto prevented the success of every effort in this country to imitate the universality with which vaccination is performed in Europe—reasons which are inherent in the nature of our political and social institutions, and which produce difficulties that can only be overcome by a radical and far-reaching change in these institutions. Before that change is undertaken, it will be surely worth while to inquire whether the game is worth the candle. It is this neglect to "think things through" to their ultimate logical consequences which vitiates the writings of so many of our most enthusiastic sanitary reformers. They are very apt to take under consideration every thing but human nature, or some other equally important factor in the problem of sanitary administration. There is something sublime about the innocence with which the average sanitarian, on discovering some great abuse which human law has failed to abate, tarries not to ask why despotic statutes have become a "dead letter," but straightway lifts up his voice, American fashion, and cries for more laws and for a more stringent despotism. This carelessness relative to personal liberty is the more inexcusable since the great principles which underlie all these vexed questions of sanitary administration have long ago been thought out and fought out on another field. But too much of our sanitary legislation has been the hastily begotten offspring of panics and emergencies; and, like all such legislation, is not adapted to the ordinary conditions of mankind. There ought to be a statute of limitations to secure the selfextinction of such dangerous laws within a definite period. But there is nothing of the kind, and our only practical mode of deliverance lies in the common sense of the community, which sooner or later, in every free country, forces injudicious enactments into a "dead letter" obscurity. Unfortunately, however, this does not remove such laws from the statute-book; consequently, whenever any little blind giant wishes to achieve a cheap notoriety by swinging a big club, he is sure to find one ready made for his hand. Here will be found a large field of usefulness for our State Boards of Health. They will do good service if they will wisely review our sanitary legislation, and will thoroughly prune away everything incompatible with reason and justice.

Considerations of this kind seem to have occupied the mind of Dr. Billings, who has contributed an essay on The Rights, Duties and Privileges of the Community in Relation to the Individual.

He expresses the opinion that the time has come for the evolution of the Sanitary Lawyer. I would suggest the need of something even higher—the Sanitary Statesman. Experience shows—and the volume now under consideration is rich in proofs of the fact—that the average doctor and the average legislator cannot be trusted with the production of sanitary laws for the community. The problem, though simple enough, seems to be beyond the grasp of the ordinary intellect. It is hard to convince your thorough-going philanthropist that with the very best of intentions he may be sowing the seed of unnumbered evils. But all history is full of similar examples; and the worst despotisms have often been the work of the best of men. These sanitarians will bear watching; and while we thank them for the good they do, they must cheerfully yield their work to criticism if they would retain the respect and the sympathy of the profession from which the greater number of them came.

